# Phase-Pure Hydroxyapatite/β-Tricalcium Phosphate Scaffolds from Ultra-Pure Precursors: Composition Governs Porosity, Strength, and SBF Kinetics

**DOI:** 10.3390/jfb16110407

**Published:** 2025-10-31

**Authors:** Panuwat Monviset, Kasama Srirussamee, Anak Khantachawana, Parichart Naruphontjirakul

**Affiliations:** 1 Biological Engineering Program, Faculty of Engineering, King Mongkut’s University of Technology Thonburi (KMUTT), Bangkok 10140, Thailand; panuwat.monv@kmutt.ac.th (P.M.); parichart.nar@kmutt.ac.th (P.N.); 2 Department of Biomedical Engineering, School of Engineering, King Mongkut’s Institute of Technology Ladkrabang (KMITL), Bangkok 10520, Thailand

**Keywords:** biphasic calcium phosphate (BCP), hydroxyapatite (HA), β-tricalcium phosphate (β-TCP), phase purity, rietveld refinement, limit of detection (LoD), simulated body fluid (SBF), freeze-dry casting, degradation kinetics

## Abstract

Biphasic calcium phosphate (BCP)scaffolds comprising hydroxyapatite (HA) and β-tricalcium phosphate (β-TCP) were produced from ultra-pure precursors and processed under an α-TCP–avoiding schedule (1100 °C, 2 h). Quantitative X-ray diffraction (Rietveld/Profex) detected no α-TCP above the ~1 wt% limit of detection and quantified post-sintering phase fractions (wt% HA/β-TCP): 99.26/0.74, 68.51/31.49, and 27.57/72.43. Across compositions, SEM/ImageJ yielded similar mean macropore sizes (≈71–80 µm), while open porosity increased with the HA fraction (27.5 ± 1.8%, 39.1 ± 2.0%, 57.1 ± 2.4%). Compressive strength decreased accordingly (1.07 ± 0.25, 0.24 ± 0.01, 0.05 ± 0.02 MPa), consistent with non-load-bearing use. In ISO-compliant simulated body fluid (28 d), medium pH remained stable (7.33–7.43); mass loss and early Ca^2+^ depletion increased with β-TCP content, consistent with more extensive surface apatite formation in β-TCP-rich scaffolds. Collectively, these data are consistent with a composition-dependent sequence—β-TCP content → densification/porosity → strength → degradation/apatite kinetics—within the tested conditions and inform parameter-based tuning of BCP scaffolds for non-load-bearing indications (e.g., alveolar ridge preservation, craniofacial void filling).

## 1. Introduction

Bone defects from trauma, disease, or surgery remain a substantial clinical challenge and continue to motivate advances in regenerative medicine. Conventional grafts— autografts and allografts—are effective but constrained by donor-site morbidity, limited supply, immune reactions, and infection risk, prompting interest in synthetic bone-graft substitutes [1,2,3]. Among these, calcium phosphate (CaP) biomaterials—especially hydroxyapatite (HA) and β-tricalcium phosphate (β-TCP)—have attracted sustained attention because they resemble bone mineral and are intrinsically osteoconductive. Moreover, β-TCP is more readily resorbed whereas HA is comparatively persistent, enabling composition-tunable behavior when combined as biphasic calcium phosphate (BCP) [4,5,6,7,8].

Clinically, BCP scaffolds have been widely adopted as bone-graft substitutes in alveolar ridge preservation, craniofacial reconstruction, and orbital floor repair, where balanced resorption and stability are essential. Their tunable degradation enables surgeons to match the material to site-specific healing rates—β-TCP-rich formulations for faster remodeling and HA-rich compositions for shape retention in long-term structural support [9,10,11]. Continuous advances in clinical translation underscore the importance of reproducible composition–property control in optimizing surgical outcomes.

Reproducible links between composition, structure, and function are often obscured by two recurring issues. First, high-temperature processing can generate unintended secondary phases (e.g., α-TCP, tetracalcium phosphate/TTCP) that alter dissolution kinetics and confound cross-study comparisons [12,13]. Second, variability in precursor quality and poorly constrained phase fractions affect densification, solubility, and ion release—even when nominally identical BCP ratios are reported; recent studies indicate that minor impurities can measurably shift β-TCP reactivity and properties [14,15]. In parts of the literature, BCP composition is reported by nominal HA/β-TCP ratios without quantitative phase verification or explicit method detection limits.

In parallel, emerging fabrication techniques such as 3D printing and doped-sintering approaches have further diversified CaP scaffold design. Additive manufacturing allows precise pore architectures and the integration of growth-factor-loaded polymers (e.g., PCL/β-TCP, GelMA/BCP composites), while dopant incorporation (e.g., Zn, Sr, Si) has been shown to enhance osteogenic response and mechanical strength [16,17,18]. However, these methods often introduce organic binders or secondary phases that obscure the intrinsic effects of HA/β-TCP ratios. The present freeze-dry-casting route therefore serves as a phase-pure baseline, isolating composition-controlled behavior free from dopant or binder interference.

To address these gaps, we adopt a precursor-engineered, phase-specific route: HA and β-TCP powders are synthesized from ultra-pure reagents, blended to defined ratios, and processed into freeze-dried scaffolds with interconnected macroporosity. A controlled post-sintering step (1100 °C, 2 h) establishes the biphasic composition while minimizing high-temperature transformations. Quantitative phase analysis by X-ray diffraction (Rietveld refinement in Profex/BGMN) reports phase fractions with uncertainties and refinement statistics, with Fourier-transform infrared spectroscopy (FTIR) as an orthogonal confirmation [19,20]. Degradation behavior is evaluated in ISO-compliant simulated body fluid (SBF; 0–28 days) alongside pH and Ca/P evolution and examined for surface apatite formation.

We test the hypothesis that phase purity—achieved from ultra-pure precursors and quantitatively verified—acts as a first-order design lever in BCP scaffolds. Specifically, we ask whether: (i) composition drift during sintering is measurable and should be explicitly reported; (ii) increasing β-TCP fraction is associated with faster degradation and earlier SBF-apatite formation, whereas HA-rich blends better retain structural stability; and (iii) uniaxial compression provides a practical benchmark of handling-level mechanical integrity for these porous architectures. By foregrounding purity-driven performance and standardizing its reporting (phase fractions with uncertainties and method LoD), this work aims to sharpen composition–structure–function relationships and offer practical guidance for tailoring HA/β-TCP scaffolds for bone regeneration.

## 2. Materials and Methods

### 2.1. Materials

HA and β-TCP powders were synthesized in-house by wet-chemical precipitation (Section 2.2). Calcium carbonate (CaCO_3_, 99.5%; Fujifilm Wako Pure Chemical Corp., Osaka, Japan) and phosphoric acid (H_3_PO_4_, 85 wt% in water; Fujifilm Wako) served as calcium and phosphate sources, respectively. Aqueous ammonia (NH_4_OH, 28–30 wt%; Kanto Chemical Co., Inc., Tokyo, Japan) was used for pH adjustment during β-TCP precipitation. For scaffold formulation, gelatin (Type B, bovine skin; Sigma-Aldrich/Merck, St. Louis, MO, USA) was used as a processing aid/binder, and hydroxyethyl cellulose (HEC; Sigma-Aldrich/Merck) as a dispersant. Ultrapure water (18.2 MΩ·cm; Milli-Q, Merck) was used throughout.

### 2.2. Preparation of HA and β-TCP Powders

HA and β-TCP were synthesized separately via a controlled wet-chemical precipitation process, each starting from 500 g of calcium carbonate (CaCO_3_, 99.5%) as the calcium source. CaCO_3_ was first calcined at 875 °C for 3 h in a ceramic crucible (2 °C·min^−1^) to yield approximately 280.15 g of calcium oxide (CaO, 5.00 mol). The CaO was then slaked in 5 L of deionized water at a CaO:H_2_O molar ratio of 1:1 to produce 370.15 g of calcium hydroxide, Ca(OH)_2_ (5.00 mol), according to:

(1) $$CaO\;\;+\;H_{2}O\to \;Ca{\left(OH\right)}_{2}$$

For HA (target Ca/P = 1.67), Ca(OH)_2_ (370.15 g; 5.00 mol) was reacted with phosphoric acid H_3_PO_4_ (85%, 345.57 g; 3.00 mol) added dropwise under continuous stirring while maintaining pH 8.5–8.9. The reaction proceeded for 2 h following:

(2) $$5[Ca({OH)}_{2}]+3{[H}_{3}{PO}_{4}]\to {[Ca}_{5}{\left({PO}_{4}\right)}_{3}\left(OH\right)]+9{[H}_{2}O]$$

For β-TCP (target Ca/P = 1.50), a second batch of Ca(OH)_2_ (370.15 g; 5.00 mol) prepared as above was reacted with H_3_PO_4_ (85%, 383.96 g; 3.33 mol). The acid was added gradually under constant stirring, and pH 6.5–7.0 was maintained by alternating additions of acid and aqueous ammonia (NH_4_OH, 28%). The mixture reacted for 2 h with real-time pH monitoring, according to:

(3) $$3[Ca({OH)}_{2}]+2{[H}_{3}{PO}_{4}]\to {Ca}_{3}{\left({PO}_{4}\right)}_{2}+6{[H}_{2}O]$$

For both syntheses, the precipitates were filtered through 5C-grade filter paper, thoroughly rinsed with deionized water, and dried overnight at 85 °C. The dried solids were lightly ground, sieved for homogeneity, and then heat-treated at 800 °C for 2 h to stabilize the initially amorphous/poorly crystalline powders prior to subsequent processing. To confirm the crystallization potential and target phase composition, representative powder aliquots were sintered separately at 1200 °C (HA) and 1100 °C (β-TCP); XRD of these sintered powders verified single-phase HA and single-phase β-TCP with no detectable secondary phases (e.g., CaCO_3_, CaO, α-TCP).

### 2.3. Preparation of BCP Scaffolds

BCP scaffolds with target HA/β-TCP ratios of 75:25, 50:50, and 25:75 (codes H75B25, H50B50, H25B75) were fabricated by freeze-dry casting. Hydroxyethyl cellulose (HEC) was dissolved in deionized water at 70 °C to obtain a clear solution; in a separate vessel, gelatin was dissolved at 40 °C. After the HEC solution cooled to <50 °C, it was combined with the gelatin solution to avoid thermal degradation of the protein. Pre-blended BCP powder was then added slowly to the mixed solution under continuous stirring to reach a 50 wt% solids loading, yielding a homogeneous slurry. The slurry was cast into molds (20 × 20 × 5 mm).

Samples were pre-frozen at −50 °C for 24 h to ensure complete solidification and then lyophilized under vacuum (condenser ≤ −50 °C; chamber ≤ 0.1 mbar) to constant mass. The freeze-dried green bodies underwent binder burnout at 600 °C in air (ramp 3 °C·min^−1^; hold 1 h) to remove gelatin/HEC, followed by sintering screening at 1100–1200 °C for 2 h (heating/cooling 5 °C·min^−1^) to develop the intended biphasic HA/β-TCP microstructure while suppressing undesired phases. Based on XRD verification, 1100 °C for 2 h was selected as the processing condition for scaffolds reported herein. For mechanical testing, sintered plates were sectioned into 15 × 15 × 3 mm specimens; the remaining material was used for microstructural and degradation analyses.

### 2.4. Characterization

#### 2.4.1. X-Ray Diffractometer (XRD)

Phases in the synthesized powders and sintered scaffolds were determined by XRD (D8 Advance, Bruker; Cu Kα, λ = 1.5406 Å; 40 kV, 25 mA). Diffraction patterns were collected from powders and gently powdered scaffold fragments over 20–60° 2θ (step size 0.01°, 0.5 s per step). Quantitative phase analysis was performed in Profex (v5.4.1; BGMN engine) using Rietveld refinement with refined background, zero shift, scale factors, unit cells, and pseudo-Voigt peak shapes; a March–Dollase preferred-orientation correction was applied where needed. ICDD reference patterns for HA and β-TCP were used (exact PDF identifiers are listed with the corresponding figures). The refinement reported Rwp and χ^2^, and the estimated limit of detection (LoD) for minor crystalline phases was ~1 wt% under these counting conditions. These analyses were used to determine the actual post-sintering HA/β-TCP phase fractions.

#### 2.4.2. Fourier Transform Infrared Spectroscopy (FTIR)

FTIR spectra were collected on an FT/IR-4600 (JASCO, Japan) in ATR mode over 400–4000 cm^−1^ at 4 cm^−1^ resolution with 32 co-added scans at room temperature. Spectra were background-subtracted and baseline-corrected prior to analysis. Band assignments for HA and β-TCP are provided in the Results.

#### 2.4.3. Scanning Electron Microscopy and Image-Based Pore Metrics

Fractured scaffold surfaces were examined by FE-SEM (JSM-7500F, JEOL, Akishima, Tokyo, Japan) after Au sputter-coating (~5 nm; 120 s). Images were acquired with SE/BSE detectors at 15 kV (typical working distance 8–10 mm) over multiple magnifications (≈200×–2000×). ImageJ software (version 1.53; National Institutes of Health, Bethesda, MD, USA) was used for pore-architecture quantification. For each composition, *n* = 3 specimens were analyzed with 10 non-overlapping ROIs per specimen. In each ROI, two orthogonal test lines were overlaid and the mean intercept length (MIL) was reported as the average pore size. Pixel-to-micrometer calibration was taken from the instrument scale bar; features smaller than ~3 µm (corresponding to ~20 pixels at the acquisition settings) were excluded A Priori to avoid counting surface cracks/charging artifacts. Values are reported as mean ± SD.

#### 2.4.4. Porosity Measurement

Bulk open porosity was determined by liquid displacement (Archimedes) using ethanol as the immersion medium to ensure wetting of interconnected pores (adapted from ceramic porosity standards such as ASTM C373/C20). Specimens were dried to constant mass (*m_dry_*), vacuum-impregnated in ethanol, then weighed saturated in air (*m_sat_*) and submerged (*m_sub_*). Per composition, *n* = 3 measurements were performed. Porosity (%) was calculated as

(4) $$Porosity\;\left(\%\right)=\frac{(m_{sat}-m_{dry})\;}{\left(m_{sat}-m_{sub}\right)}\times 100$$

#### 2.4.5. Compression Test

Uniaxial compression was conducted on plate-like rectangular specimens (15 × 15 × 3 mm) using an Instron 5848 equipped with a calibrated 10 kN load cell. Specimens were placed between flat compression platens and loaded with the 3 mm thickness along the loading axis at a crosshead speed of 0.50 ± 0.05 mm·min^−1^ under ambient laboratory conditions. Engineering stress was computed from the instantaneous force divided by the initial cross-sectional area (15 × 15 mm^2^), and compressive strength was taken as the peak stress prior to catastrophic failure. For each composition, *n* = 3 replicates were tested; results are reported as mean ± SD.

### 2.5. In Vitro Degradation Test in Simulated Body Fluid (SBF)

Scaffolds were accurately weighed (W_0_) and immersed in 30 mL of SBF, prepared according to ISO 23317:2014 [21], with a pH of 7.4 and an ionic strength of 0.16 M (see Table 1 for detailed ion composition). The immersion was carried out at 37 °C under static conditions, sealed conditions without medium renewal, under static, to avoid mechanically induced erosion and to mimic a passive in vivo degradation environment. Samples were incubated for a total period of 28 days, and weight measurements were taken at defined intervals (days 0, 7, 14, 21, and 28). At each time point, three scaffolds from each composition group were retrieved, freeze-dried, and reweighed (W_t_) to assess degradation. The percentage of weight loss was calculated using the following equation:

(5) $$Degradation\;\left(\%\right)=\;\frac{(W_{0}-Wₜ)\;}{W_{0}}\;\times \;100$$

Additionally, changes in the micro-morphology and pore structure of the degraded scaffolds were examined using SEM.

### 2.6. Ion Release Test

Ion release from the BCP scaffolds was assessed by measuring the concentrations of Ca^2+^ ions released into the SBF medium at each predetermined time point. Similarly to the degradation test (0, 7, 14, 21, and 28 days). After each incubation interval, the media were collected, thoroughly mixed to ensure homogeneity, and analyzed using Inductively Coupled Plasma Optical Emission Spectroscopy (ICPS-9800 Series, Shimadzu, Kyoto, Japan).

### 2.7. Statistical Analysis

Data are reported as mean ± SD with individual data points shown. Analyses used specimen-level means (ROI-level measurements were averaged to one value per specimen). For one-factor comparisons among compositions (H25B75, H50B50, H75B25) at each time point we used: one-way ANOVA with Tukey’s HSD when assumptions (normality, homoscedasticity) held, and Welch’s ANOVA with Games–Howell pairwise tests when the homogeneity-of-variance assumption was violated (applied to compressive strength). The familywise error rate was controlled within each figure panel. Exact n and the test used are stated in each caption. All tests were two-sided with α = 0.05. Analyses were performed in MATLAB R2025b. Significance notation used across figures: **** *p* < 0.0001; *** *p* < 0.001; ** *p* < 0.01; * *p* < 0.05; ns, not significant.

## 3. Results and Discussion

### 3.1. Characterizations of HA and β-TCP Powders

**Figure 1:**
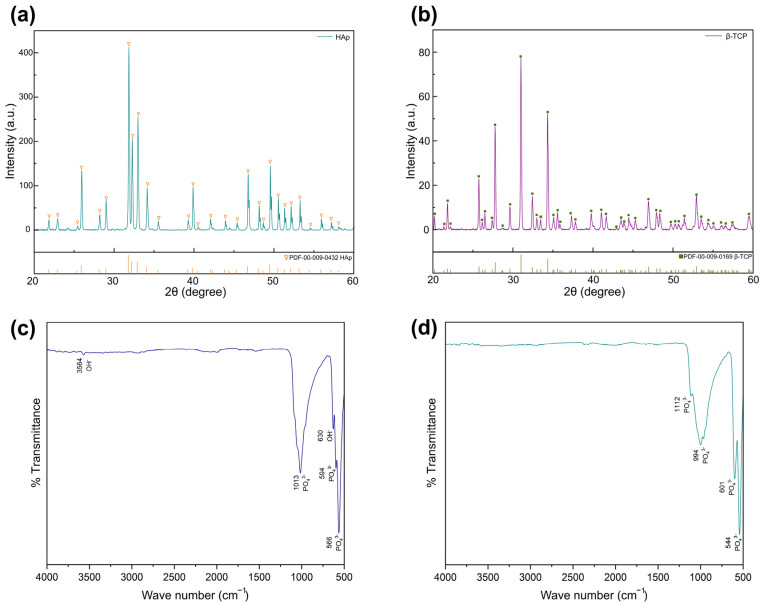
(**a**) XRD pattern of phase-pure HA with reference ticks (ICDD PDF 00-009-0432). (**b**) XRD pattern of β-TCP (ICDD PDF 00-009-0169). (**c**) ATR-FTIR spectrum of HA showing characteristic PO_4_^3−^ and OH^−^ bands. (**d**) ATR-FTIR spectrum of β-TCP with characteristic PO_4_^3−^ bands.

Before blending into BCP, we verified the phase identity and purity of the individually synthesized HA and β-TCP powders by XRD and FTIR (Figure 1). The XRD patterns (Figure 1a,b) match the reference cards for HA (ICDD PDF 00-009-0432) and β-TCP (ICDD PDF 00-009-0169), with no crystalline secondary phases detected above the XRD method LoD under our counting conditions. FTIR spectra (Figure 1c,d) corroborate these assignments—showing, for HA, the PO_4_^3−^ ν_1_ near ~960 cm^−1^, the ν_4_ doublet at ~565/603 cm^−1^, and OH-related features at ~630/3570 cm^−1^; and for β-TCP, PO_4_^3−^ bands at ~947–975 cm^−1^, shoulders at ~1030–1045 and ~1115–1125 cm^−1^, and bends at ~545–552 and ~597–607 cm^−1^ [22,23,24]. These results confirm that the phase-specific precursors are single phase and suitable for downstream BCP fabrication. Subsequent sections report the actual HA/β-TCP fractions after scaffold sintering and analyze composition drift and degradation behavior.

### 3.2. Characterization and Morphology of BCP Scaffolds

**Figure 2:**
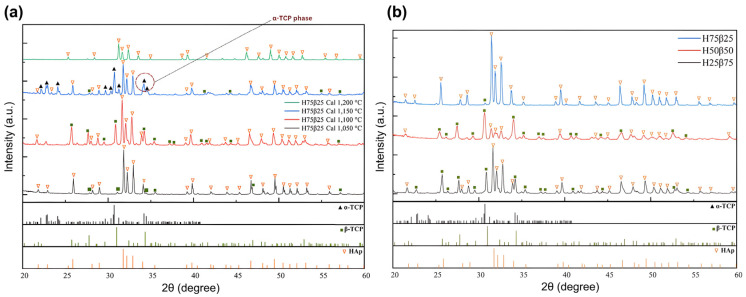
XRD screening and post-sintering phase composition of BCP scaffolds. (**a**) Temperature screening of the H75B25 blend sintered at 1050–1200 °C (2 h), highlighting the onset of α-TCP above ~1120 °C; reference ticks for HA, β-TCP, and α-TCP are shown. (**b**) XRD patterns of H25B75, H50B50, H75B25 scaffolds sintered at 1100 °C/2 h with reference ticks (ICDD).

XRD screening of the H75B25 blend across 1050–1200 °C (Figure 2a) defined a sintering window that preserves the biphasic HA/β-TCP structure while suppressing undesired transformations. At 1050 °C, β-TCP reflections are weaker/broader, consistent with the minor β-TCP fraction and/or reduced crystallinity at sub-optimal temperatures. At ≥1150 °C, additional reflections attributable to α-TCP appear, in line with the canonical β → α transition near ~1120–1125 °C and reports of HA dehydroxylation/phase evolution above ~1000–1200 °C [25,26,27]. On this basis, 1100 °C/2 h was selected. Under these conditions, the three blends (H25B75, H50B50, H75B25) exhibit HA and β-TCP patterns with no α-TCP detected above the XRD LoD (Figure 2b). Rietveld refinement (Profex/BGMN) yields the post-sintering fractions (Table 2): H25B75 = 27.57 wt% HA/72.43 wt% β-TCP; H50B50 = 68.51/31.49; H75B25 = 99.26/0.74 (Rwp 20–24%, χ^2^ 2.1–4.0). The measured drift from nominal—most pronounced for the HA-rich blend—is consistent with prior observations that BCP ratios can shift during heat treatment near ~1100 °C unless schedules are carefully engineered [28].

### 3.3. Mechanical Properties of BCP Scaffolds

**Figure 3:**
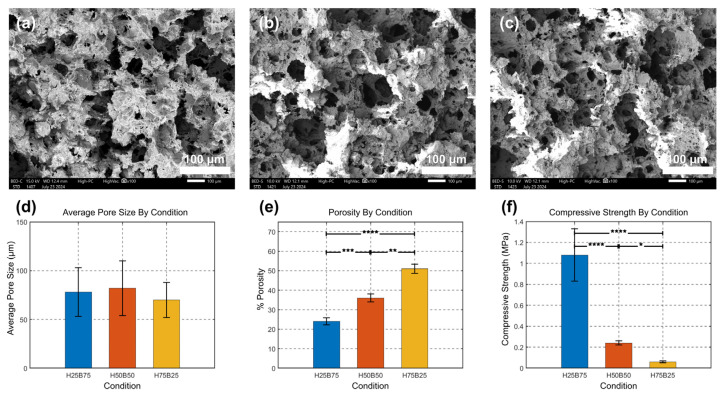
Microstructure and mechanical properties of BCP scaffolds sintered at 1100 °C for 2 h. (**a**–**c**) SEM (100×; scale bar = 100 μm) of H25B75, H50B50, and H75B25. (**d**) Average pore size from ImageJ line-intercept stereology (≥30 pores per specimen averaged to one value; *n* = 3 specimens per composition). (**e**) Open porosity (%) by Archimedes (ethanol displacement; adapted from ASTM C373/C20; *n* = 3). (**f**) Compressive strength (MPa; *n* = 3). Data are mean ± SD. Panels (**d**–**e**): one-way ANOVA (Tukey–Kramer). Panel (**f**): Welch’s ANOVA with Games–Howell pairwise tests. α = 0.05. Significance coding as defined in Section 2.7. Significance notation: **** *p* < 0.0001; *** *p* < 0.001; ** *p* < 0.01; * *p* < 0.05.

SEM at 100× reveals a similar, interconnected macroporous architecture across all compositions (Figure 3a–c), with no gross collapse. Quantitatively, mean pore size did not differ among groups (Figure 3d): 79.31 ± 42.71 μm (H25B75); 80.99 ± 39.76 μm (H50B50); 67.89 ± 31.43 μm (H75B25) (Mean ± SD, *n* = 3 specimens per composition; ≥30 pores/specimen averaged). One-way ANOVA: F(2, 6) = 0.10, *p* = 0.903; Tukey–Kramer, all pairwise comparisons ns; In contrast, open porosity increased with the HA fraction (Figure 3e): 27.5 ± 1.8% (H25B75) < 39.1 ± 2.0% (H50B50) < 57.1 ± 2.4% (H75B25) (*n* = 3). One-way ANOVA: F(2, 6) = 154.01, *p* = 6.98 × 10^−6^. Tukey–Kramer: H50B50 vs. H25B75 (*p* = 0.00118; **), H75B25 vs. H50B50 (*p* = 0.000103; ***), H75B25 vs. H25B75 (*p* = 5.70 × 10^−6^; ****). These trends are consistent with distinct sintering responses: β-TCP densifies effectively at 900–1100 °C, whereas HA generally requires 1200–1450 °C for extensive densification [29,30]. Accordingly, we fixed the schedule at 1100 °C for 2 h (from the temperature screening in Figure 2a) to remain below the β-TCP → α-TCP transition and preserve phase stability.

Mechanical results follow this microstructural context (Figure 3f). The β-TCP-rich scaffold (H25B75) exhibited the highest compressive strength (1.07 ± 0.25 MPa), the balanced composition (H50B50) was intermediate (0.24 ± 0.01 MPa), and the HA-rich scaffold (H75B25) was the weakest (0.05 ± 0.02 MPa) (*n* = 3). Welch’s ANOVA indicated a composition effect (*p* < 0.01); Games–Howell pairwise tests identified H25B75 > H50B50 (*p* < 0.0001; ****), H25B75 > H75B25 (*p* < 0.0001; ****), and H50B50 > H75B25 (*p* < 0.0245; *). With similar mean pore sizes but markedly different porosities—and Rietveld-verified phase fractions decreasing in β-TCP from H25B75 to H75B25 (Table 2)—we attribute the strength differences primarily to densification/porosity, with β-TCP enabling better neck growth at 1100 °C while HA remains relatively undersintered at this temperature. All strengths lie within the non-load-bearing range typical for porous BCP scaffolds.

### 3.4. Degradation Studies

#### 3.4.1. Medium Stability & Gross Morphology in SBF

**Figure 4:**
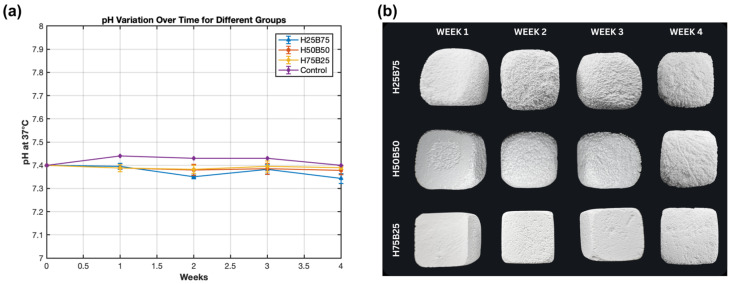
Medium stability and macroscopic morphology during SBF immersion. (**a**) pH vs. time at 37 °C in SBF for H25B75, H50B50, H75B25, and an SBF-only control (mean ± SD; *n* = 3). (**b**) Weekly macroscopic images (weeks 1–4) showing preserved geometry and composition-dependent surface roughening.

Across 4 weeks in SBF at 37 °C, the medium pH for all groups—including the SBF-only control—remained within a narrow window (≈7.33–7.43; Figure 4a), in line with ISO-style SBF at pH ≈ 7.40 and with reports that test parameters (e.g., solution formulation/circulation) influence readouts [31]. During week 1, H50B50 and H75B25 showed small transient dips, consistent with initial ion exchange and carbonate equilibration; thereafter, H25B75/H50B50 displayed slight upticks but stayed within the same range, and H75B25 returned to the baseline trajectory—behavior expected for supersaturated SBF prepared/handled per ISO.

Macroscopically, all scaffolds preserved their overall geometry over time (Figure 4b), while surface roughening progressed in a composition-dependent manner—more evident for the β-TCP-rich H25B75, moderate for H50B50, and least for the HA-rich H75B25—consistent with the higher solubility of β-TCP relative to HA and the well-described dissolution–reprecipitation pathway in BCPs. These observations point to controlled scaffold–medium interactions and earlier surface reactivity for β-TCP-rich compositions, setting the stage for the quantitative degradation kinetics (mass loss and Ca^2+^ balance) reported in Section 3.4.2.

#### 3.4.2. Degradation Kinetics in SBF

**Figure 5:**
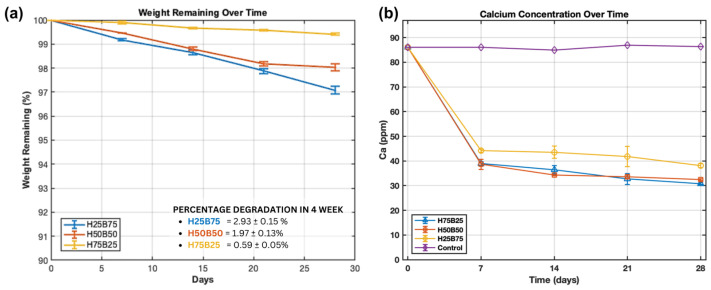
Degradation kinetics in SBF at 37 °C under static, sealed conditions without medium renewal. (**a**) Weight remaining (%) vs. time for H25B75, H50B50, and H75B25 (mean ± SD; *n* = 3). Y-axis zoomed to 90–100% for clarity. (**b**) SBF Ca concentration (mg·L^−1^) vs. time, including an SBF-only control, showing an early drop followed by a plateau consistent with surface precipitation processes.

Consistent with the pH stability and composition-dependent surface roughening in Figure 4, the quantitative SBF kinetics over 28 days at 37 °C show that mass loss scales with the β-TCP fraction (Figure 5a): H25B75 degrades fastest (2.93 ± 0.15%), H50B50 is intermediate (1.97 ± 0.13%), and H75B25 is slowest (0.59 ± 0.05%). In parallel, the SBF Ca concentration drops sharply during week 1 and then plateaus (Figure 5b), with the largest net decrease for H25B75, intermediate for H50B50, and the smallest for H75B25, whereas the control remains comparatively flat. Taken together—under near-physiological pH and static, sealed SBF—these coupled trends (greater mass loss plus stronger Ca^2+^ depletion in β-TCP-rich scaffolds) are consistent with composition-dependent dissolution followed by reprecipitation/apatite nucleation on the more soluble β-TCP surfaces (with possible carbonate co-precipitation). The ordering matches the Rietveld-verified phase fractions in Table 2 (β-TCP: 72.43 > 31.49 > 0.74 wt%) and explains the macroscopic roughening in Figure 4 under ISO-style SBF exposure.

#### 3.4.3. SEM Analysis of BCP Scaffold Surface

**Figure 6:**
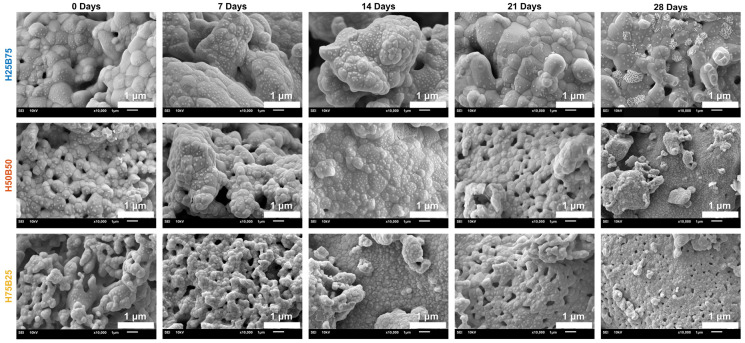
High-magnification SEM time-series (10,000×; 37 °C SBF, ISO-23317) at 0, 7, 14, 21, and 28 days. Rows show compositions (H25B75, H50B50, H75B25); columns show immersion time. By day 28, plate/needle-like apatite domains (circled) form a near-continuous layer on H25B75, patchy coverage appears on H50B50, and sparse islands remain on H75B25. Scale bars as indicated; images are representative (*n* = 3 specimens per composition, ≥3 ROIs per time point).

High-magnification SEM (10,000×) tracked surface evolution over 0–28 days in SBF (Figure 6). At day 0, all groups displayed granular sintered surfaces characteristic of the starting ceramics. By day 7, the β-TCP–rich H25B75 already showed scattered nano-globules; H50B50 exhibited fewer nuclei; H75B25 was largely unchanged. During days 14–21, H25B75 developed cauliflower-like aggregates and coalescing spherulitic features, while H50B50 showed moderate nucleation with partial coalescence; H75B25 presented isolated nodules on regions of bare substrate. At day 28, H25B75 displayed near-continuous coverage by plate/needle-like crystallites consistent with SBF-induced apatite; H50B50 showed patchy but appreciable coverage; H75B25 retained sparse islands with substantial exposed surface. This temporal and composition-dependent sequence mirrors the mass-loss and Ca^2+^ consumption trends (Figure 5) and is consistent with faster dissolution followed by surface precipitation on β-TCP–rich compositions under our static SBF conditions.

Taken together, across the three BCP compositions studied and a fixed, α-TCP–avoiding schedule (1100 °C/2 h) verified by XRD–Rietveld, our observations—considered alongside recent literature—support a composition-led cascade: increasing β-TCP → greater densification/lower open porosity → higher compressive strength → faster early-stage SBF conversion/apatite formation. At 1100 °C, β-TCP–rich blends densify more readily than HA, whereas HA typically requires ~1200–1300 °C to achieve comparable densification [32]; meanwhile, exceeding ~1120 °C risks the β → α-TCP transition, motivating the present processing window. Under ISO-style SBF, early Ca depletion and time-dependent surface crystallites on β-TCP–rich scaffolds are consistent with solution-mediated apatite precipitation and should be interpreted as a screening indicator rather than proof of in vivo bonding [33]. All measured strengths (≤1.07 MPa) lie well below commonly reported trabecular-bone compressive strengths (order-of-magnitude MPa range), placing these freeze-dried BCPs squarely in the non-load-bearing regime [34,35]. Finally, the tendency for higher β-TCP fractions to accelerate dissolution/reprecipitation while HA-rich blends better retain geometry aligns with the greater solubility of β-TCP vs. HA and with composition-dependent biological response, while recognizing that true in vivo resorption is also cell-mediated [36]. Within these bounds (single architecture, static SBF, one firing schedule), a purity-centered workflow—with explicit Rietveld phase fractions and reporting of the method detection limit—should improve reproducibility and enable predictable, composition-driven tuning of HA/β-TCP scaffolds for non-load-bearing indications [37].

## 4. Conclusions

Quantitatively verified phase-pure BCP scaffolds show that increasing β-TCP (at 1100 °C/2 h) reduces open porosity, raises compressive strength (up to 1.07 MPa), and accelerates SBF conversion, whereas HA-rich blends trade strength for dimensional stability. All measured strengths (≤1.07 MPa) are far below commonly reported trabecular-bone compressive strengths (order-of-magnitude MPa range, varying with density/anatomical site), reinforcing a non-load-bearing assignment for these freeze-dried BCPs. The processing window deliberately avoids the β → α-TCP transition and the trends observed are consistent with contemporary evidence on HA densification and TCP phase evolution. Overall, this delivers a practical composition–property map for tailoring HA/β-TCP scaffolds (β-TCP-rich for faster conversion; HA-rich for shape retention) and provides a phase-pure baseline for future studies introducing dopants, alternate schedules, or architected porosity.

From a clinical perspective, β-TCP-rich scaffolds are most suitable for rapid-remodeling or defect-filling applications, such as alveolar ridge preservation, socket grafting, and small cancellous defects, where faster resorption and ionic exchange can stimulate osteogenesis. Conversely, HA-rich scaffolds are better suited for sites requiring long-term dimensional stability and shape retention, including craniofacial contour augmentation, orbital floor, and maxillofacial reconstruction, where gradual resorption and persistent scaffold structure are advantageous.

These results bridge fundamental composition-controlled behavior with clinical indication, offering a purity-driven rationale for tailoring HA/β-TCP ratios in future bone-repair scaffolds.

## Figures and Tables

**Table 1:** Ionic composition of SBF prepared according to ISO 23317:2014.

Ion	Concentration (mM)	Compound Source
Na^+^	142.0	NaCl
K^+^	5.0	KCl
Mg^2+^	1.5	MgSO_4_·7H_2_O
Ca^2+^	2.5	CaCl_2_·2H_2_O
Cl^−^	147.8	NaCl, KCl, CaCl_2_·2H_2_O
HCO_3_^−^	4.2	NaHCO_3_
HPO_4_^2−^	1.0	K_2_HPO_4_
SO_4_^2−^	0.5	MgSO_4_·7H_2_O

**Table 2:** Rietveld-quantified phase fractions of sintered BCP scaffolds at 1100 °C/2 h.

BCP Scaffolds	HA (wt%)	β-TCP (wt%)	α-TCP (wt%)	Rwp (%)	χ^2^
H25B75	27.57	72.43	<LoD	22.29	2.10
H50B50	68.51	31.49	<LoD	24.37	3.98
H75B25	99.26	0.74	<LoD	20.30	4.03

Notes: values from single-pattern Rietveld refinements in Profex/BGMN; LoD ≈ 1 wt% (3σ) for minor crystalline phases under these counting conditions.

## Data Availability

The original contributions presented in the study are included in the article, further inquiries can be directed to the corresponding author.

## Abbreviations

The following abbreviations are used in this manuscript:

α-TCP	Alpha-tricalcium phosphate
β-TCP	Beta-tricalcium phosphate
APC	Article processing charge
ATR	Attenuated total reflectance
BCP	Biphasic calcium phosphate
BGMN	Rietveld refinement engine/software “BGMN”
BSE	Backscattered electron
CaP	Calcium phosphate
DOI	Digital object identifier
FE-SEM	Field-emission scanning electron microscopy
FTIR	Fourier transform infrared
GBR	Guided bone regeneration
HA	Hydroxyapatite
HEC	Hydroxyethyl cellulose
ICDD	International Centre for Diffraction Data
ICP-OES	Inductively coupled plasma optical emission spectroscopy
ISO	International Organization for Standardization
LoD	limit of detection
MIL	Mean intercept length
PDF (ICDD)	Powder Diffraction File (ICDD)
Rwp	Weighted-profile R-factor
ROI	Region of interest
SBF	Simulated body fluid
SD	Standard deviation
SE	Secondary electron
SEM	Scanning electron microscopy
TTCP	Tetracalcium phosphate
XRD	X-ray diffraction
